# High-throughput interaction screens illuminate the role of c-di-AMP in cyanobacterial nighttime survival

**DOI:** 10.1371/journal.pgen.1007301

**Published:** 2018-04-02

**Authors:** Benjamin E. Rubin, TuAnh Ngoc Huynh, David G. Welkie, Spencer Diamond, Ryan Simkovsky, Emily C. Pierce, Arnaud Taton, Laura C. Lowe, Jenny J. Lee, Scott A. Rifkin, Joshua J. Woodward, Susan S. Golden

**Affiliations:** 1 Division of Biological Sciences, University of California San Diego, La Jolla, CA, United States of America; 2 Department of Microbiology, University of Washington, Seattle, WA, United States of America; 3 Center for Circadian Biology, University of California San Diego, La Jolla, CA, United States of America; University of Wyoming, UNITED STATES

## Abstract

The broadly conserved signaling nucleotide cyclic di-adenosine monophosphate (c-di-AMP) is essential for viability in most bacteria where it has been studied. However, characterization of the cellular functions and metabolism of c-di-AMP has largely been confined to the class Bacilli, limiting our functional understanding of the molecule among diverse phyla. We identified the cyclase responsible for c-di-AMP synthesis and characterized the molecule’s role in survival of darkness in the model photosynthetic cyanobacterium *Synechococcus elongatus* PCC 7942. In addition to the use of traditional genetic, biochemical, and proteomic approaches, we developed a high-throughput genetic interaction screen (IRB-Seq) to determine pathways where the signaling nucleotide is active. We found that in *S*. *elongatus* c-di-AMP is produced by an enzyme of the diadenylate cyclase family, CdaA, which was previously unexplored experimentally. A *cdaA*-null mutant experiences increased oxidative stress and death during the nighttime portion of day-night cycles, in which potassium transport is implicated. These findings suggest that c-di-AMP is biologically active in cyanobacteria and has non-canonical roles in the phylum including oxidative stress management and day-night survival. The pipeline and analysis tools for IRB-Seq developed for this study constitute a quantitative high-throughput approach for studying genetic interactions.

## Author summary

Cyclic di-adenosine monophosphate (c-di-AMP) is a molecule that has significant roles in many microorganisms. This work shows the existence of c-di-AMP for the first time in photosynthetic microorganisms, cyanobacteria, and demonstrates its role in survival during the light-to-dark shifts that occur in day-night cycles. Despite the obvious importance of adaptation to these daily cycles for organisms that are fundamentally reliant on light, such as cyanobacteria, understanding of diurnal physiology is lacking because most cyanobacterial research is conducted during growth in constant light. To identify other players in c-di-AMP’s function we developed a low-cost and efficient method for finding interactions between genes. The technique combines one mutation, in this case for the gene that encodes the enzyme for synthesis of c-di-AMP, with thousands of other individual mutations to find pairwise interactions that affect fitness of the resulting mutants. Mutants are tagged with DNA barcodes to allow their survival to be easily tracked in a population of cells. The method enables us to place the function of c-di-AMP within the context of pathways previously known to be involved in day-night survival. Taken together, this work expands the known roles of c-di-AMP, improves our understanding of cyanobacterial survival in day-night cycles, and presents an improved approach for determining genetic interactions.

## Introduction

The signaling nucleotide cyclic di-adenosine monophosphate (c-di-AMP) has been implicated in a wide range of biological processes since its discovery less than a decade ago [1]. The molecule is active in multiple pathways including potassium transport, regulation of central metabolism, cell wall homeostasis, gene expression, and DNA damage responses [2]. In addition, c-di-AMP is the only second messenger that is essential to most of the organisms in which it is studied [3]. Despite the biological importance of c-di-AMP, *in vivo* studies of this signaling nucleotide have focused on a narrow array of Firmicutes and to a lesser extent Actinobacteria. Expanding the investigation of c-di-AMP to a broader diversity of microbial phyla may reveal new functions for this molecule.

Organisms in the photosynthetic phylum Cyanobacteria are key players in global carbon, oxygen, and nitrogen cycling, and are promising platforms for renewable production of industrial chemicals [4–6]. While c-di-AMP has not been reported in the phylum, the existence of enzymes and riboswitches predicted by homology to interact with the molecule suggests its presence [2,7–9]. Signaling nucleotides previously studied in cyanobacteria (cGMP, c-di-GMP, (p)ppGpp, and cAMP) serve important roles in environmental responses and have revealed activities specific to photosynthetic organisms, such as regulating phototaxis, photosystem repair, and dark survival [8,10]. Characterization of c-di-AMP metabolism and its role in cyanobacteria may similarly lead to a better understanding of the phylum, as well as the functions of c-di-AMP.

C-di-AMP research, as a young field with many unknowns, has particularly benefited from untargeted genetic interaction screens. Screens focused on secondary mutations that relieve the phenotypes of c-di-AMP-related mutants (alleviating interactions) have successfully identified genetic interactions with c-di-AMP synthases and phosphodiesterases [11,12]. However, these screens have been limited to assaying positive interactions, were non-quantitative, and have been performed under only a single condition.

Quantitative genetic interaction screens to identify aggravating as well as alleviating interactions would provide a powerful tool to elucidate c-di-AMP function. Such screens have been conducted for other processes using next-generation sequencing paired with transposon mutagenesis (Tn-Seq) [13–15]. Tn-Seq for genetic interactions relies upon the generation of a new mutant library for each screen in the background of a knockout of interest [16]. While informative, the need to generate and characterize a new transposon-mutant library for each interaction screen is both costly and labor intensive [17]. These features have limited the applicability of Tn-Seq for interaction screens. A variation on Tn-Seq, RB-TnSeq, provides a cheaper and less laborious approach for screening transposon mutant libraries, in which the fitness of mutants in the population is tracked by sequencing and counting of 20-base pair “barcodes” in each transposon [18]. This approach has not yet been applied to interaction screens, where it offers great potential for mitigating their logistical weaknesses and enlarging their scope.

Here, we used genetic and biochemical approaches to elucidate the presence and roles of c-di-AMP in the cyanobacterium *Synechococcus elongatus* PCC 7942. To further probe the molecule’s functions we developed and implemented an approach we named interaction RB-TnSeq, or IRB-Seq, to quantitatively measure genetic interactions with the c-di-AMP cyclase under multiple conditions.

## Results

### Presence of c-di-AMP and its cyclase

The putative c-di-AMP-producing and degrading enzymes in *S*. *elongatus* were identified computationally. Two domain organizations, DHH-DHHA1 (PF01368 and PF02272) and 7TM-7TMR_HD (PF07698), have been found to degrade c-di-AMP [19]. The software package HMMER [20] identified one of each in *S*. *elongatus*. Based on homology, the DHH-DHHA1 domain-containing protein, RecJ (Synpcc7942_1886), likely serves as a single-stranded DNA exonuclease. However, it may also act as a non-specific phosphodiesterase for c-di-AMP and linear 5′-phospho diadenylate (pApA), as documented in the literature [19]. The 7TM-TMR_HD-containing protein, Synpcc7942_0779, is a reciprocal best hit with the *Listeria monocytogenes* c-di-AMP degrading enzyme PgpH, with homology focused around the HD domain active site. Therefore, Synpcc7942_0779, and to a lesser extent RecJ, are likely phosphodiesterases for c-di-AMP in *S*. *elongatus*.

**Fig 1:**
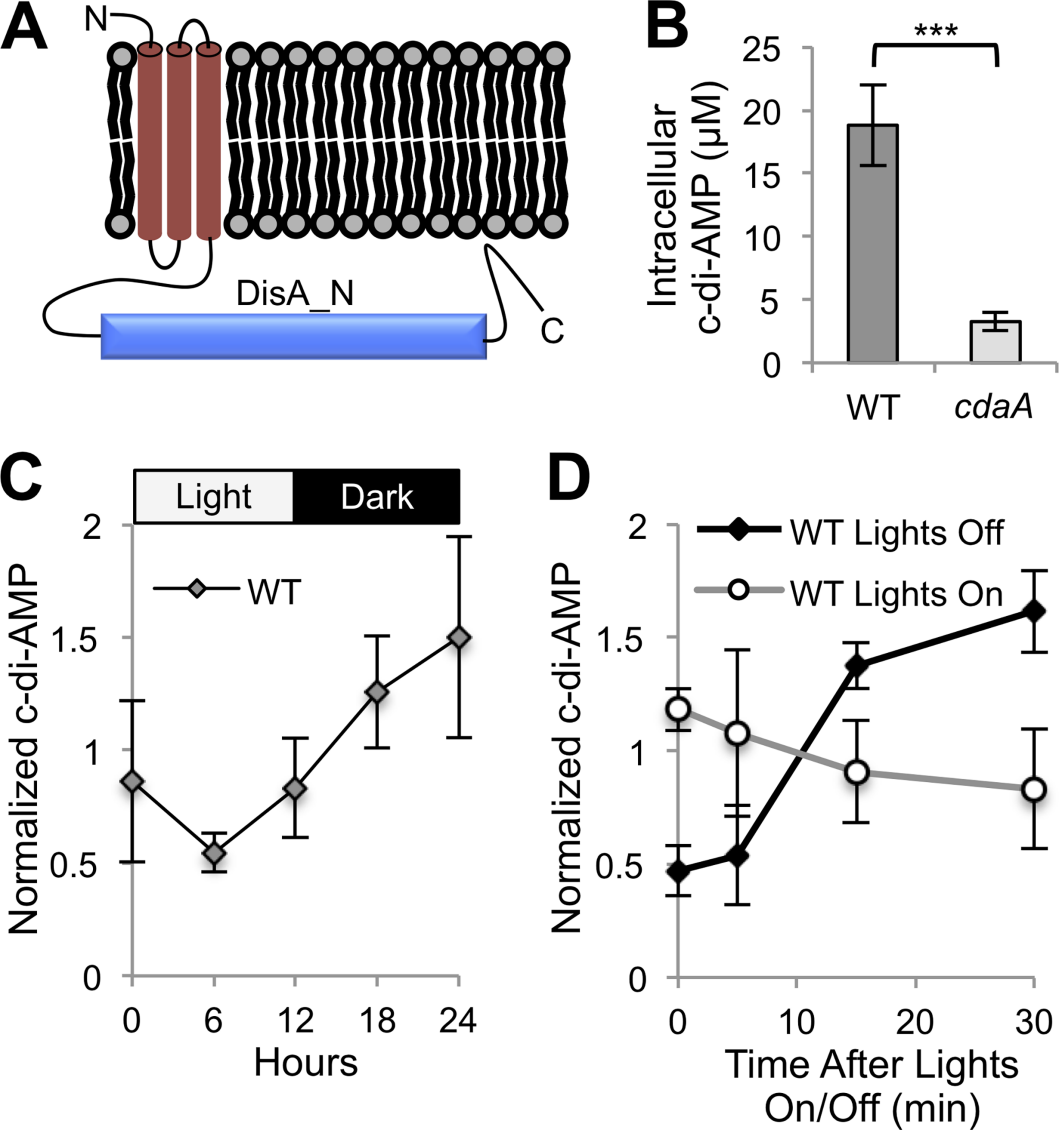
Presence, synthesis, and light-dependence of c-di-AMP in *S*. *elongatus*. (*A*) Predicted CdaA protein topology. (*B*) Intracellular c-di-AMP measured by LC-MS for WT and *cdaA* transposon mutant (8S16-L9). Intracellular c-di-AMP concentrations were determined from raw quantities using cell volume (see Materials and Methods). The error bars represent standard error (SE) of five time points taken throughout a 24-h LDC in quadruplicate. ***P < 10^−7^ (Mann-Whitney-Wilcoxon Test). (*C*) C-di-AMP quantities in WT over one LDC. (*D*) C-di-AMP quantities upon the onset of darkness or light in WT. (C and D) C-di-AMP quantities are normalized by dividing by the average c-di-AMP concentration of the replicate. Error bars represent SE of four replicates.

A single protein in the organism contains a DisA_N domain (PF02457), currently the only domain known to be responsible for c-di-AMP production [21]. Although presence of this putative synthase has been noted several times in cyanobacteria, it has yet to be experimentally validated [2,8,9,22]. This sequence, annotated at time of publication as “protein of unknown function DUF147”, predicts three transmembrane segments and a cytoplasmic DisA_N domain (Fig 1*A*). The arrangement of domains identifies the protein as a member of the most abundant family of DisA_N domain-containing proteins, CdaA [2,23]. Thus, we refer to this previously unannotated gene, which encodes the putative cyclase of c-di-AMP, as *cdaA* (Synpcc7942_0263).

To validate this bioinformatic prediction of the c-di-AMP cyclase, we quantified c-di-AMP levels in wild type (WT) and a *cdaA*-null mutant using LC-MS. The WT value of 18.8 μM (Fig 1*B*) is higher than reported in other organisms, including *Staphylococcus aureus* [24] and *Bacillus subtilis* [25], where the nucleotide plays biologically important roles. While c-di-AMP is essential for many other c-di-AMP-producing organisms, a fully segregated insertion mutant for *cdaA*, which has no other paralog in the genome of *S*. *elongatus*, is viable under constant-light standard laboratory conditions (S1 Fig). The residual signal in the *cdaA* mutant, potentially attributable to c-di-AMP (Fig 1B), was within the background noise in mass spectrometry quantification and unlikely to represent presence of the molecule (S2 Fig). In further support of CdaA’s role in c-di-AMP synthesis, the *S*. *elongatus cdaA* was expressed in *Escherichia coli*, which does not naturally produce c-di-AMP; high levels of the molecule were detected (S3 Fig). These data strongly suggest that *S*. *elongatus* produces c-di-AMP, and that CdaA catalyzes its synthesis.

Concentrations of other signaling nucleotides studied in cyanobacteria change in response to light [8]. To investigate whether this may be the case with c-di-AMP in *S*. *elongatus*, we sampled every six hours over one 12-h light:12-h-dark cycle (LDC). The c-di-AMP concentration was variable in WT both among samples within time points and between them, with an apparent, although non-significant, trend upwards at nighttime (Fig 1*C*). Given that signaling nucleotide responses to light have been shown to occur within minutes in cyanobacteria [26], we performed higher resolution sampling around the light-to-dark and dark-to-light transitions. While light did not have a large or immediate effect on c-di-AMP, a three-fold increase in the nucleotide’s levels was observed 15 minutes after onset of darkness (Fig 1*D*).

### LDC sensitivity in *cdaA* mutant

**Fig 2:**
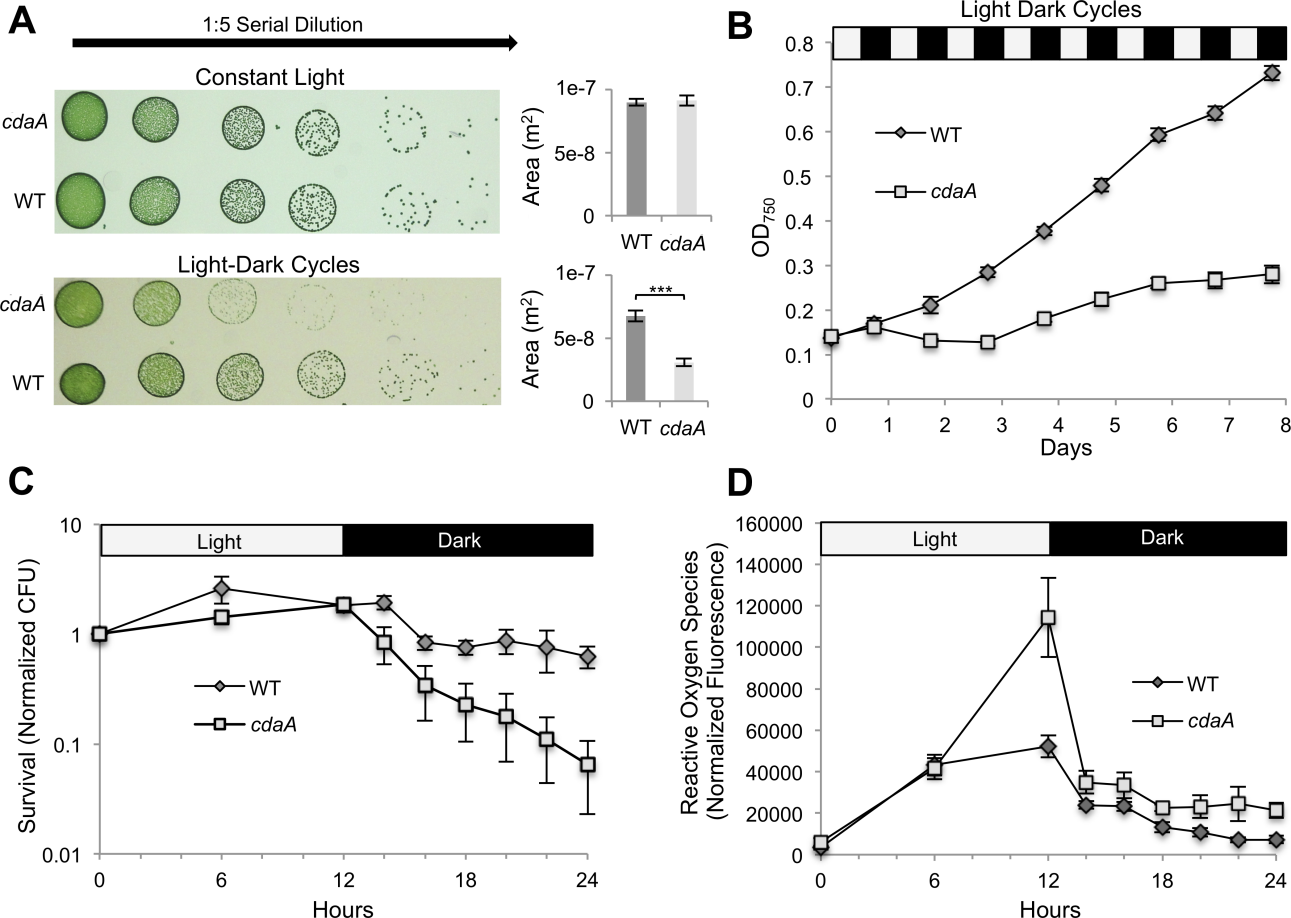
Sensitivity of *cdaA* mutant to LDCs. (*A*) Growth of WT and *cdaA* transposon mutant (8S16-L9) measured by spot plate under constant light and LDCs. ***P < 10^−5^ (Mann-Whitney-Wilcoxon Test). (*B*) Growth curve of WT and *cdaA* mutant in liquid culture in bioreactors under LDCs. (*C*) High resolution measurement of survival of WT and *cdaA* mutant throughout one LDC. Survival is quantified by CFU present at each time point normalized to CFU present at the first time point for each replicate. (*D*) ROS was measured by H2DCFDA fluorescence normalized by OD_750_. Error bars in all figure parts indicate SE of four replicates.

Based on the observed increase in c-di-AMP levels upon onset of darkness, as well as previous research showing the importance of the closely linked signaling nucleotide ppGpp to dark survival in *S*. *elongatus* [10], we examined whether c-di-AMP is necessary to survive LDCs. In both solid and liquid media, the *cdaA* mutant has decreased growth in LDCs, but not in constant light (Fig 2*A* and 2*B*). This phenotype was successfully complemented by the addition of a WT copy of the *cdaA* gene to a neutral site (S4 Fig). Furthermore, although the *cdaA* mutant’s absorbance spectrum was not significantly different from WT’s before sensitizing LDCs, bleaching was apparent 30 h after the start of an LDC, and worsened thereafter (S5 Fig). Therefore, the *cdaA* mutation and the associated decrease of c-di-AMP levels are likely responsible for the LDC-specific deleterious phenotype.

To explore the nature and cause of the LDC sensitivity, we examined populations over one LDC. While the *cdaA* mutant grew similarly to WT over the light portion of the LDC, viability of the mutant, as measured by colony forming units in outgrowths in continuous light from specific time points, rapidly decreased upon the onset of darkness. This difference was significant after two hours of exposure to darkness (P < .05; *t* test), and became more pronounced over the course of the night (Fig 2*C*). Recent work showed that reactive oxygen species (ROS) correlate with death in darkness for *S*. *elongatus* [27]. Indeed, reactive oxygen species peaked in the mutant after the onset of darkness at more than two fold the level in WT and remained higher than WT through the course of the night (P < 10^−5^; Mann-Whitney-Wilcoxon Test) (Fig 2*D*). These data suggest that a still-cryptic mechanism actively kills the *cdaA* mutant specifically in the dark stage of the LDC and potentially as a consequence of high oxidative stress.

### Genetic interactions of c-di-AMP

#### IRB-Seq design

**Fig 3:**
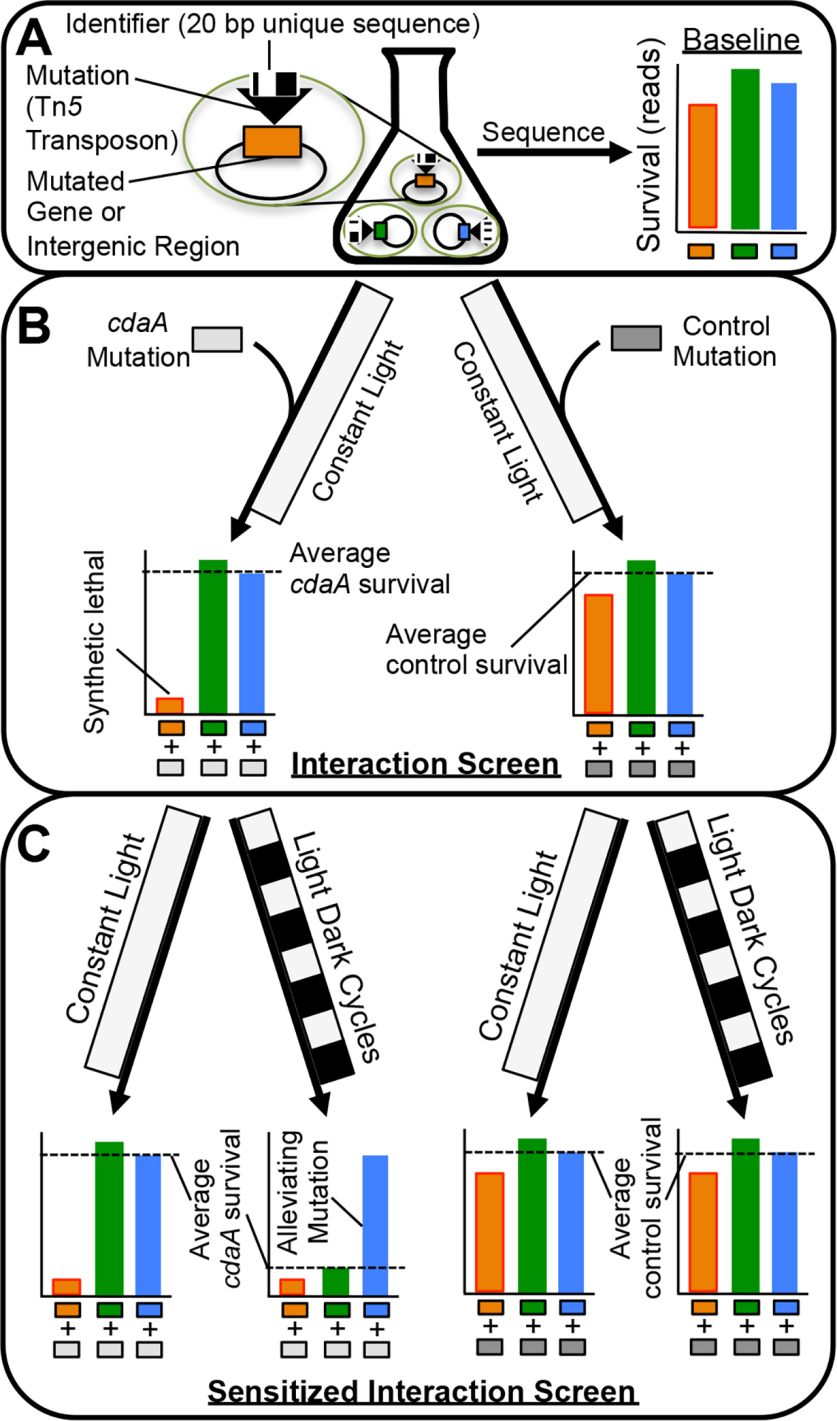
IRB-Seq approach to genetic interaction screens. (*A*) Each mutant in the starting library contains a loss-of-function mutation with a unique identifier sequence (barcode) that has been previously linked to the mutation’s locus. After the archived library is thawed the barcodes present in each mutant are sequenced using next-generation sequencing to determine their baseline levels. (*B*) The library is then split into two aliquots with one receiving an experimental mutation (AM5403 for this screen), and one receiving a control mutation with no expected fitness costs (AM5329 for this screen). After outgrowth, these two aliquots are sequenced for barcode quantification and compared, which allows identification of genetic interactions between the experimental mutation and the preexisting barcoded mutations. (*C*) The double mutant library is grown under a condition of stress for the experimental mutation and a four-way comparison between it and controls for genotype and stress condition enables identification of genetic interactions.

The c-di-AMP signaling pathway is still enigmatic in cyanobacteria with no *in vivo* evidence that identifies other members of the pathway. Therefore, we used an unbiased genome-wide approach to identify genes involved in c-di-AMP function through genetic interactions with c-di-AMP’s cyclase, CdaA. The approach relied on a previously developed dense transposon insertion mutant library in *S*. *elongatus* [28]. Briefly, this library was built with the RB-TnSeq method [18], in which every loss-of-function mutant in the library contains a unique identifier sequence, or barcode (Fig 3*A*). By using next-generation sequencing of barcodes the survival and relative fitness of the approximately 150,000 barcoded mutants in the library can be tracked under control as well as experimental conditions. In this way, the *S*. *elongatus* RB-TnSeq library can be used for pooled, quantitative, whole-genome mutant screens.

In interaction RB-TnSeq (IRB-Seq), the survival of library mutants is observed under a genetic perturbation instead of or in addition to the environmental perturbations normally used in RB-TnSeq. In practice, a second mutation is added to all RB-TnSeq library members; this procedure combines the second mutation with every other individual mutation in the library so that the fitness impact of each double mutant can be determined. In this case, the second mutation inactivated *cdaA* and conferred a different antibiotic resistance than the transposons used to construct the original mutant library (see Materials and Methods). As a control for changes to the library caused by the addition of a second mutation that are not specific to *cdaA* mutation, a non-deleterious mutation was added to a separate aliquot of the initial library. The experimental and control double-mutant libraries were then grown under dual selection so that every member of the library also contained the secondary mutation of interest (*cdaA* or the control). At this point the barcodes of the two double-mutant libraries were sequenced and compared (Fig 3*B*). These data were used to determine genetic interactions, or instances in which the fitness effects of the library mutation and the *cdaA* mutation are not simply additive (see Materials and Methods). These interactions could in turn be used to identify relationships between genes. In addition, because of the *cdaA* mutant’s decreased viability in LDCs, we sensitized our library of double mutants by exposure to LDCs (Fig 3*C*). Frequencies of the barcodes after this stress were compared to a genotypic and environmental control (see Materials and Methods). In this way IRB-Seq enabled a quantitative assay of genetic interactions with *cdaA*, under both control and sensitizing environmental conditions.

**Fig 4:**
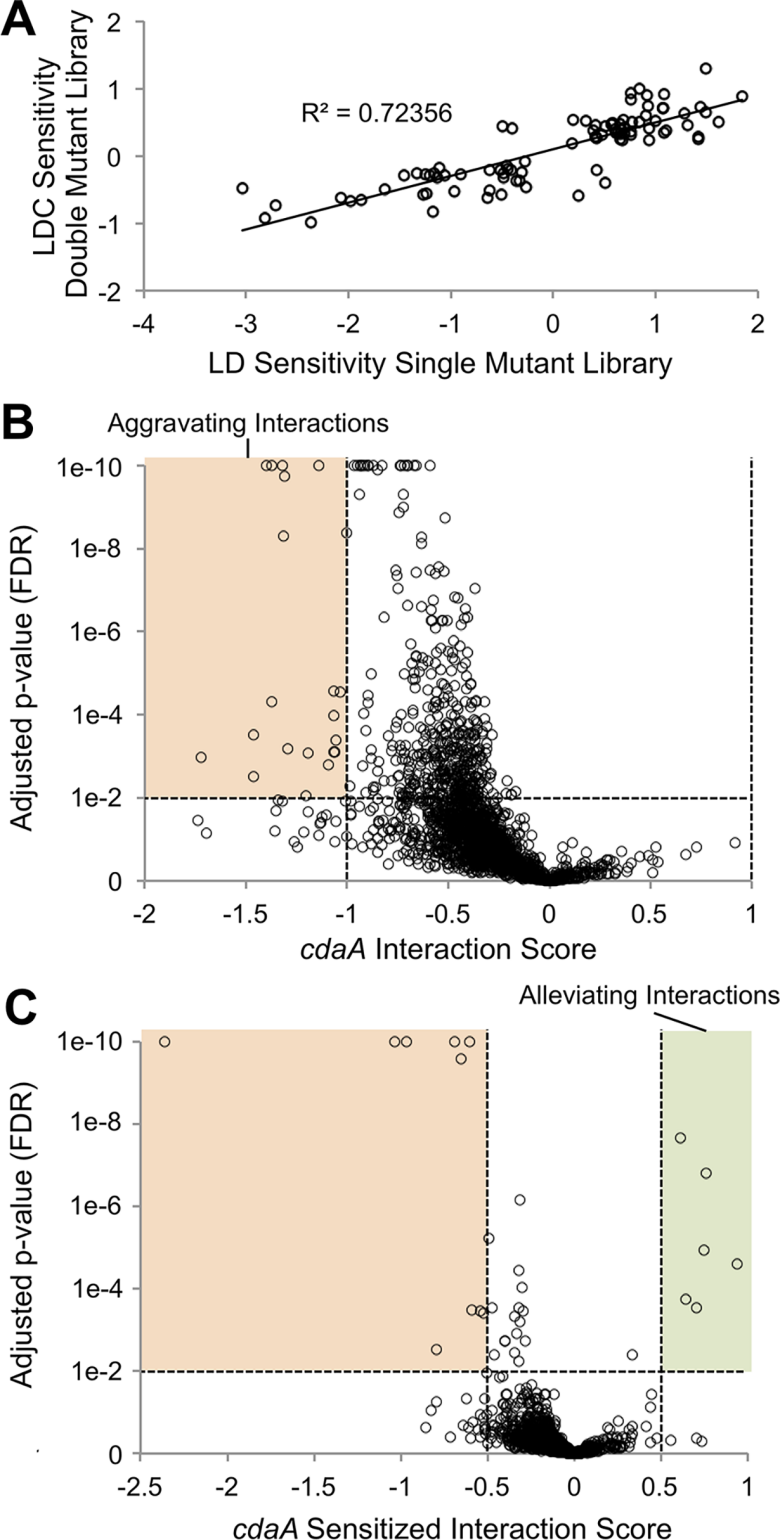
*cdaA* genetic interactions using IRB-Seq. (*A*) Validation of double-mutant screening by comparison to the previous single-mutant library LDC-sensitivity screen. Each circle represents a gene’s score for LDC sensitivity from a previously conducted screen of LDCs on the single-mutant library (x-axis), compared to a similar screen conducted here on a double-mutant library that carries a control mutation (y-axis). A linear regression analysis was used to determine correlation. (*B* and *C*) Plots of (*B*) genetic interactions and (*C*) LDC-sensitized genetic interaction of library genes with *cdaA*. Genes above the horizontal dashed line have FDR<0.01 (Linear mixed-effects model). Absolute values of interaction score thresholds of (*B*) 1 or (*C*) 0.5 are indicated by vertical dashed lines. All points with FDR<10^−10^ are plotted as FDR = 10^−10^.

Before attempting IRB-Seq we ensured that the double-mutant library retained sufficient diversity for meaningful functional screens. Of the original 154,949 barcoded mutants in the library, we were able to recover, on average, 100,180 (65%) after addition of the second mutation. This number still represents approximately 30 insertion mutants for the average gene. To further test efficacy of the double-mutant library for screens we used the version containing the control mutation to reproduce the results of a previous screen for LDC-sensitive genes [29]. The screen of the control double-mutant library was conducted under different light conditions than that of the previous single-mutant library screen, and in flasks instead of bioreactors, but nevertheless the two screens strongly correlate (r = 0.85; Fig 4*A*). It is of note that the slope of the regression of the double-mutant screen on the single-mutant screen is approximately 0.4, suggesting that, although the relative phenotypes were similar, the mutants did not diverge as much over the course of the double-mutant experiment. The described changes in the experimental conditions could account for this difference. Based on these results we carried out IRB-Seq using a *cdaA* deletion mutation added to the *S*. *elongatus* RB-TnSeq library.

#### IRB-Seq interaction screen

The first genetic interactions examined were those that are detectable under standard laboratory conditions upon the addition of the *cdaA* mutation to the library (Fig 3*B*). As expected, given that the *cdaA* mutant shows no fitness defects under control conditions (Fig 2*A*), all of the strong genetic interactions (FDR < 0.01, genetic interaction score absolute value > 1) were negative (aggravating or synthetic interactions) (Fig 4*B*, S1 Dataset). Such synthetic interactions can result from redundancy in the function associated with the two mutated genes. Two of the top annotated synthetic interactions with *cdaA* were with genes that encode flavoproteins Flv1 (Synpcc7942_1810) and Flv3 (Synpcc7942_1809), which form a heterodimer that allows the release of high-energy electrons from photosystem I to oxygen without producing reactive oxygen species. Although these proteins are dispensable under standard laboratory conditions, they become important for decreasing oxidative stress and allowing growth under variable conditions such as fluctuating light [30,31]. Also in the top ten annotated synthetic interactions were genes that encode a 6-pyruvoyl-tetrahydropterin synthase-like protein potentially involved in a pathway important for resisting UV-A stress (Synpcc7942_1184) [32–34], a glutaredoxin-related protein (Synpcc7942_1145), and Psb28 (Synpcc7942_1679), a protein involved in repair of photosystem II in related cyanobacteria [35]. These interactions suggest that, in the absence of the functional *cdaA*, a cell under increased oxidative stress (Fig 2*D*) becomes more dependent on proteins that offer electron sinks or protection against reactive oxygen species.

We used targeted loss-of-function mutants to validate the genetic interactions between oxidative stress-related genes and *cdaA* observed through IRB-Seq. A double mutant of *cdaA* and the oxidative stress-related gene with the strongest aggravating interaction, *flv1*, was grown in in liquid culture in competition with the *cdaA* single mutant. The double mutant showed drastically decreased survival in comparison with the *cdaA* single mutant (S6*A* Fig), as expected based on the IRB-Seq results. This effect was much greater than that of the single *flv1* mutant (S6*B* Fig), suggesting a real aggravating interaction in the double mutant. The results support *cdaA’s* interaction with oxidative stress as well as the ability of IRB-Seq to identify genetic interactions.

Another synthetic interaction apparent from the screen under standard laboratory conditions is with potassium transport. The fifth-ranked aggravating interaction candidate is the K^+^ transport protein, TrkH, encoded by *synpcc7942_1080*. Its genomic neighbor, *synpcc7942_1081*, is the second-ranked aggravating interactor. Although Synpcc7942_1081 is unannotated, it is predicted to contain a TrkA domain (PF02080) [36], a complex partner of TrkH [37]. Therefore, the *cdaA* mutant is likely sensitized to defects in potassium import.

Given the genetic interaction with potassium transport, we also tested for physical interaction of c-di-AMP with a potassium transporter. TrkH and Synpcc7942_1081 do not have homologs known to bind c-di-AMP. However, another potassium transport-related polypeptide in *S*. *elongatus*, KdpD (Synpcc7942_1729), has been found in *S*. *aureus* to bind c-di-AMP [38,39]. KdpD is a sensor kinase involved in regulation of the Kdp potassium transport system. DRaCALA, a method based upon differential diffusion of bound and unbound radiolabelled c-di-AMP across a nitrocellulose membrane [40], indicates binding of c-di-AMP to *S*. *elongatus* KdpD (S7 Fig). This finding supports the physiological importance of c-di-AMP in potassium homeostasis in *S*. *elongatus*.

#### IRB-Seq sensitized interaction screen

When the *cdaA* double mutant library was sampled under sensitizing LDCs (Fig 3*C*), we again observed synthetic interactions, but now also observed a number of positive (alleviating interactions) (Fig 4*C*, S2 Dataset). Alleviating interactions can occur when the affected genes are in the same pathway and their individual and combined effects are equivalent, or when the mutations counteract each other. The presence of synthetic interactions in the sensitized screen can be explained as genes whose loss exacerbates the moderate survival impact of the *cdaA* mutation in LDCs. For the sensitized interaction screen, we decreased the interaction score for consideration to greater than absolute value of 0.5. This change was based on our observation that the selection strength of the LDC screening conditions enabled the differentiation of genuine, but subtle, mutant phenotypes (Fig 4*A*). Among the six alleviating candidates for *cdaA* in LDCs (FDR < 0.01, genetic interaction > 0.5) four are proteins involved in macromolecule degradation. The cell wall recycling protein MurQ (Synpcc7942_2577), the top hit among alleviating interactions, may be explained by decreased cell wall turnover being permissive in the *cdaA* mutant, which has been associated with disruption of cell wall homeostasis in multiple species [2]. The reason for the more general enrichment of genes that encode degrading enzymes among alleviating mutations is unclear.

The fifth-ranked hit among suppressors and the strongest hit among the synthetic interactions were circadian clock component CikA (Synpcc7942_0644) and circadian-involved sigma factor RpoD2 (Synpcc7942_1746), respectively. Mutants of *cikA*, the phosphatase for the master clock transcription factor RpaA, leave RpaA in a highly phosphorylated state [41]. This RpaA state locks the clock into activating nighttime processes and the detoxification of oxidative stress [27,42,43]. The single *cikA* mutant has a slightly positive effect on light-dark survival and has been shown to be capable of alleviating another LDC-sensitive mutant, *kaiA* [29]. Therefore, it might be expected that the *cdaA* mutant, which is unable to fully clear oxidative stress and survive the night, would be suppressed by *cikA*. The *rpoD2* gene, which has by far the strongest synthetic interaction with *cdaA* in the sensitized screen, encodes a sigma factor that causes changes in circadian rhythms when mutated [44]. However, the broader transcriptional and physiological effects of this mutation are unknown, and when the interaction was tested with targeted loss-of-function mutations, it was not supported (S8 Fig). It is unclear whether this result is due to a technical shortcoming in the follow-up experiment, a difference in the behavior of the *rpoD2-cdaA* double mutant in the mixed library population and in a two-member competition, or an inaccurate sensitized interaction call by IRB-Seq. Therefore, the presence and nature of an interaction of *cdaA* with the circadian clock is yet uncertain.

## Discussion

These investigations present the use of classical molecular biology and the newly developed IRB-Seq approach to characterize c-di-AMP in the model cyanobacterium, *S*. *elongatus*. The molecule exists in *S*. *elongatus* at concentrations that are likely physiologically relevant. It is synthesized by the product of the previously unannotated gene *synpcc7942_0263* (assigned here as *cdaA*). One important physiological function of CdaA appears to be survival through LDCs, which is impaired in the *cdaA* mutant (Fig 2). This finding suggests a role for c-di-AMP as an intracellular signal of LDCs. Finally, through the use of IRB-Seq, we determined the interaction landscape of c-di-AMP, which links the signaling nucleotide to oxidative stress management and potassium transport. This work expands our understanding of the intracellular signaling mechanisms in *S*. *elongatus*, illuminates new roles of c-di-AMP, and describes a high-throughput approach for identifying genetic interactions.

### The non-essential nature of c-di-AMP in *S*. *elongatus*

C-di-AMP controls many aspects of bacterial physiology and is essential in the bacterial phylum Firmicutes, where most research on the molecule has taken place. The essential nature of c-di-AMP has been connected to its role in central metabolism [45], its linkage to levels of another signaling nucleotide (p)ppGpp [11], and its role in potassium homeostasis [46]. Importantly, the absence of c-di-AMP is lethal in Firmicutes in rich media, whereas minimal media are permissive for a null mutant. A possible explanation for *S*. *elongatus’* viability with the *cdaA* mutation is that, as a photoautotroph, it is far more independent of nutrients in the medium than the previously studied heterotrophs. Regardless of its cause, the essential nature of c-di-AMP in many organisms has limited the ability to study this nucleotide [21]. Future genetic exploration of *cdaA* will be facilitated by the viability of the *S*. *elongatus cdaA* mutant.

### The IRB-Seq approach to genetic interaction screens

The IRB-Seq approach developed for this study enables high-throughput quantitative genetic interaction screens with minimal sequencing prep. In the approach, a second mutation is added directly to an existing RB-TnSeq library, removing the need of previous approaches to recreate a library in a new background and re-determine all insertion loci for each screen (Fig 3) [13–15]. Traditional sequencing preparation used in classical Tn-Seq studies is also avoided because survival of mutants is quantified by PCR and sequencing of 20 bp “barcodes” present in each transposon which serve as identifiers for each clone in the mutant library (BarSeq) [18]. IRB-Seq requires only one PCR and ~1/100^th^ of an Illumina HiSeq 4000 lane per sample to provide a genome-wide quantitative measure of genetic interactions ranging from strong alleviation to full synthetic lethal. The advantage of a quantitative alternative to previous suppressor screens used in c-di-AMP research [11,12] is particularly apparent in this study. The *cdaA* mutant’s lack of fitness phenotype under constant light, and moderate phenotype under sensitizing LDCs, would have made traditional suppressor screens difficult, and likely ineffective. In addition, the high-throughput nature of this approach to genome-wide genetic interaction screens makes it feasible to conduct IRB-Seq screens in replicate and under many different sensitizing and permissive conditions.

### Potential causes of c-di-AMP spike at night

While this work does not determine the mechanism of the CdaA mediated c-di-AMP increase after darkness, several possibilities can be considered in light of the literature. One potential explanation is a transcriptional increase around dusk of c-di-AMP’s cyclase, CdaA, potentially controlled by the circadian clock. However, the immediate change in c-di-AMP level upon onset of darkness (Fig 1*D*), combined with a lack of dusk- or dark-induced expression of *cdaA* [47–49], make circadian or transcriptional control unlikely. Nor does the quantity of CdaA's substrate, ATP, increase in darkness [50,51], and thus is not likely to explain the increases in c-di-AMP. Therefore, it is more likely that the spike in c-di-AMP level results from dark-dependent changes in the activity of CdaA or c-di-AMP’s phosphodiesterase.

### Illuminating the role of c-di-AMP in nighttime survival

Elucidating the survival of Cyanobacteria in LDCs is important for improved understanding of both a phylum of tremendous ecological importance and an environmental challenge relevant to all photosynthetic organisms. For ease of research, however, most experiments on cyanobacteria have been conducted in simplifying constant-light conditions. The work in LDCs that exists shows oxidative stress management as a key component for surviving LDCs [27,29]. Similarly to the LDC-sensitive circadian clock mutants of *rpaA* and *kaiA*, lethality in the *cdaA* mutant occurred specifically upon the onset of night following high oxidative stress at dusk (Fig 2). The death in the mutant begins concurrent with a spike in c-di-AMP level in the WT (Fig 1*D*). This correlation suggests a role for the molecule in the day-night transition, a seemingly crucial period for surviving LDCs in the mutants where it has been studied. This role is further supported by the finding that a number of the top *cdaA* synthetically interacting genes determined by IRB-Seq are involved in oxidative stress mitigation. Thus, c-di-AMP appears to be involved in LDC survival through management of oxidative stress, a characteristic shared with LDC-sensitive clock mutants.

A possible basis for the *cdaA* mutant’s sensitivity to LDCs and oxidative stress may be potassium transport. C-di-AMP has previously been associated with multiple potassium transporters [2]. In *S*. *aureus* c-di-AMP negatively regulates the Kdp potassium uptake system through its binding to the histidine kinase KdpD [38]. In this study KdpD was suggested by DRaCALA to bind c-di-AMP in *S*. *elongatus*. Additionally, two proteins from the Trk family of potassium transporters (Synpcc7942_1080 and Synpcc7942_1081) were found among the top synthetic interactions, suggesting that the *cdaA* mutant is sensitized to potassium transport mutations. Altered potassium transport previously has been shown in cyanobacteria to sensitize the cells to the oxidative stress-producing conditions of high light and heavy metal exposure [52,53]. Therefore, the canonical role of c-di-AMP in potassium homeostasis may, in *S*. *elongatus*, be involved in the non-canonical function of LDC survival through oxidative stress regulation.

### Future uses of IRB-Seq

IRB-Seq is an inexpensive and straightforward approach to high-throughput quantitative interaction screens and is suitable for addressing an array of questions in different organisms. With 25 published RB-TnSeq libraries [28,54] and many more under development, there exists ample starting material for IRB-Seq screens, assuming the ability to deliver a second mutation into the host with high efficiency. In this case, natural transformation was sufficient to generate the double-mutant library, but other techniques such as conjugation may be necessary for species that are not naturally competent. The experimental pipeline and analysis tools developed here make this assay feasible for genetic interactions of any gene of interest. Furthermore, the addition of multiple mutations into the library, or reporters paired with cell sorting, should enable screening for more complex genetic interactions as well as identification of effects on gene expression by genetic perturbations.

## Materials and methods

### Strains and culture conditions

WT in this study and the background for all mutants was *Synechococcus elongatus* PCC 7942, stored in our lab as AMC06. For all experiments, growth was conducted in BG-11 media with appropriate antibiotics at 30°C [55]. Unless otherwise indicated, liquid culture growth was under constant light in 250 mL flasks containing 100 mL of culture shaken at 150 rpm (Thermo Fisher MaxQ 2000 Orbital Shaker).

### Targeted mutants

For mutagenesis by transformation, standard *S*. *elongatus* protocols [55] were altered for dark-sensitive mutants. The typical overnight incubation in darkness after DNA addition was instead performed under 70 μmol photons·m^−2^·s^−1^. For experiments using *cdaA* loss-of-function mutants, with the exclusion of IRB-Seq and IRB-Seq validation experiments, the plasmid for insertion mutagenesis (8S16-L9, conferring kanamycin (Km) resistance) was from the previously published unigene set [56]. For IRB-Seq and IRB-Seq validation experiments, which required a loss-of-function strain with an antibiotic resistance other than Km, a *cdaA*-deletion plasmid (AM5403) carrying the spectinomycin- (Sp) and streptomycin- (Sm) resistance gene *aadA* was constructed using CYANO-VECTOR procedures and devices [57]. This deletion removed a region from 8 bp upstream to 575 bp downstream of the translation start site of *cdaA*.

### *cdaA* LDC characterization

Phenometrics ePBR photobioreactors (version 1.1; Phenometrics Inc.) were used in LDC liquid culture assays for measurement of: survival, OD_750_, c-di-AMP quantity, and ROS. The bioreactors were inoculated with 400 mL of culture at OD_750_ = 0.1. They were then grown under 150 μmol photons·m^−2^·s^−1^ with filtered air bubbled in at a rate of 50 mL/min. After 12 h, the bioreactor cultures were re-diluted to OD_750_ = 0.1 and exposed to a 12:12 h LDC for purposes of low-light entrainment of the circadian clock; dark-sensitive mutants were previously shown to survive at this light intensity in the bioreactors [27]. After this entrainment cycle the light intensity was increased to the restrictive condition of 500 μmol photons·m^−2^·s^−1^ for the remaining LDCs, at which point assays for physiology were conducted.

High-resolution measurement of survival, c-di-AMP quantity, ROS, and absorbance spectrum were all conducted immediately following the increase of the bioreactors’ LDC light intensity to 500 μmol photons·m^−2^·s^−1^. Survival was measured using a 1:5 dilution series over 8 wells with the first well containing a 1:10 dilution of the bioreactor culture. The 8 dilutions were plated in 4 μL spots on solid medium and incubated under continuous illumination at 150 μmol photons·m^−2^·s^−1^ for 5 d, at which point they were photographed. The photographs were then analyzed for colony forming units (CFUs) and for surface area of the colonies using ImageJ [58]. For quantification of c-di-AMP, 1.5 mL of culture was sampled, pelleted at 4 C at 20,000 g for 3 min, and resuspended in 500 μL methanol with 20 μL of 0.5 μM heavy-labeled c-di-AMP. The mixture was then stored at -20 C until quantification (See C-di-AMP quantification). ROS was quantified on 1 mL of culture using the fluorescent marker H_2_DCFDA (Life Technologies catalog no. D399) as described previously [27]. Absorbance spectra were taken using a Tecan Infinite M200 plate reader.

LDC-specific sensitivity assays were also conducted on plates. A 1:5 dilution series over 8 wells was made for *cdaA* and a WT with the first well containing culture at OD_750_ = 0.02. These dilutions of *cdaA* and a WT were then plated in 4 μL spots on duplicate plates. One of these plates was grown under 125 μmol photons·m^−2^·s^−1^ constant light for 5 d, while the other was grown under 125 μmol photons·m^−2^·s^−1^ LDCs for 8 d. Although LDCs at this light intensity are permissive in the bioreactors, they constitute a restrictive condition for dark-sensitive mutants on plates, where there is no self-shading by the culture.

### C-di-AMP biochemical and proteomic quantification

#### C-di-AMP quantification

Samples stored at -20°C for c-di-AMP quantification (from: *cdaA* LDC Characterization) were pelleted, resuspended in 50 μL of 0.5 μM heavy-labeled c-di-AMP, and then mixed with 500 μL of methanol and sonicated. After centrifugation of lysed cells, the supernatant fraction was collected. The remaining pellet was resuspended in 50 μL of H_2_0, mixed with 500 μL of methanol, and centrifuged again to collect a second supernatant fraction. Both methanol fractions were pooled and evaporated, and the final pellet containing c-di-AMP was resuspended in 50 μL of double distilled H_2_O. Mass spectrometry analysis was performed as previously described [9]. Intracellular concentrations of c-di-AMP were determined using the cell volume of 2.47 e^-10^ L, which was estimated using length 2.512 μm and width 1.118 μm [59].

#### C-di-AMP binding assay

Binding validation was conducted with radiolabeled c-di-AMP using the DRaCALA approach as previously described [9]. Protein candidates identified as interacting with c-di-AMP sepharose beads were expressed from pET20b and pMAL vectors in *Escherichia coli* BL21. Following induction, *E*. *coli* cell lysates were incubated with radiolabeled c-di-AMP, spotted on nitrocellulose membrane, and visualized with a Typhoon imager (GE Healthcare).

### IRB-Seq

#### Interaction screen

To look for genetic interactions of *cdaA* with the mutated loci in the previously created RB-TnSeq library [28], the library was transformed with a *cdaA* deletion mutation plasmid (AM5403) and separately a Neutral Site I control mutation plasmid (AM5329). An aliquot of the *S*. *elongatus* RB-TnSeq library was first thawed as previously described [28]. Before transformation, 4 T_0_ samples were taken with the equivalent of 10 mL at OD_750_ = 0.3, and were pelleted and frozen at −80°C. Transformation was conducted in triplicate with the *cdaA* deletion vector and the control vector (6 transformations total) using the modified transformation protocols described above in which no dark incubation was included. Both plasmids contained Sp and Sm resistance cassettes to allow selection in the Km resistant background of the RB-TnSeq library. To maintain the diversity of the 1.52·10^5^-member library, we attempted to plate a total of approximately 5·10^5^ mutants over 10 plates for each replicate of Δ*cdaA* (4.38·10^5^ average/replicate mutants achieved) and control (5.31·10^5^ average/replicate mutants achieved) double-mutant libraries (60 plates total). After growth for 4 d in 140 μmol photons·m^−2^·s^−1^ under Km, Sp, and Sm selection, plates were harvested and combined into 6 tubes (one for each replicate and experimental condition). At this point two samples for each tube, equivalent to 10 mL at OD_750_ = 0.3, were pelleted and frozen at −80°C. These samples served as both the post-selection samples for the interaction screen and the T_0_ samples for the sensitized interaction screen. Because direct transformation was used to introduce the *cdaA* deletion and control mutation, any barcoded mutants that disable transformation were not represented in the resulting double-mutant libraries.

#### Sensitized interaction screen

The tubes containing the harvested interaction screen plates were used as inocula for the LDC-sensitized interaction screen. Flasks containing 100 ml BG-11 for each replicate and genotype were inoculated in duplicate at OD_750_ = 0.01 (12 flasks total). The duplicates were then split into constant light and 12:12 h LDCs. Both light conditions were conducted under 30 μmol photons·m^−2^·s^−1^ for the first 24 h period as entrainment for the LDC flasks, after which light intensity was increased to 150 μmol photons·m^−2^·s^−1^ for all flasks, a condition determined to be restrictive for dark-sensitive mutants. Once the cultures had grown to approximate OD_750_ of 0.64 (6 generations), two 5 mL samples for each were pelleted and frozen at −80°C. These samples would be compared to the T0 samples taken after the double-mutant library was harvested from plates (See Interaction screen). All samples from the interaction screens had genomic DNA extracted [55], and survival of constituent mutants quantified, using the previously developed BarSeq protocol [18].

#### Interaction screen analysis

To estimate genetic interactions, the survival of library constituents containing the *cdaA* mutation were compared to those containing the control mutation. A similar analysis had been conducted previously with the untransformed library to identify differential survival of the single mutants in the presence and absence of LDCs [29]. The current analysis was conducted identically, except we considered the Δ*cdaA* background of the library to be the experimental condition and the control mutation background to be the control condition. As in Welkie et al. [29], we removed 24,868 barcodes falling outside of annotated genes and 27,763 barcodes that were not within the middle 80% of genes. We also removed any gene represented by fewer than three barcodes in different positions. This curation resulted in 102,136 barcodes distributed across 1,961 genes remaining for further analysis.

To avoid errors when log-transforming the read counts, we added a pseudocount of one to the number of reads for a barcode for each sample. We then divided this value by the total number of reads in the sample calculated before any pruning, and log-2 transformed this sample-normalized number of reads.

In the experiment a single starting pool (T_0_) of barcoded strains was split into 3 control and 3 experimental samples. The log-2 count for a barcode from the starting pool was subtracted from the values for the 6 derived samples. We then discarded any gene without at least 15 reads in the starting pool and summed across barcodes before adding the pseudocount (41/1961), leaving 1920 genes and 101,876 barcodes.

To determine an interaction score for each gene, we used R [60] to fit a pair of nested linear mixed effects models to the sample- and read-normalized log-2 transformed counts using maximum likelihood (S3 Dataset):

$$y_{i,j,k}=\mu_{g}+T_{j}+B_{i}+\varepsilon_{i,j,k};B_{i}∼iid\;N\left(0,\varsigma_{g}{}^{2}\right);\varepsilon_{i,j,k}∼iid\;N\left(0,\sigma_{g}{}^{2}\right)$$ (1)

$$y_{i,j,k}=\mu_{g}+B_{i}+\varepsilon_{i,j,k};B_{i}∼iid\;N\left(0,\varsigma_{g}{}^{2}\right);\varepsilon_{i,j,k}∼iid\;N\left(0,\sigma_{g}{}^{2}\right)$$ (2)

where *y*_*i*,*j*,*k*_ is the normalized log-2 value for barcode *i* in gene *g* with genetic background *j* for sample *k*, *μ*_*g*_ is the average value for the gene, *T*_*j*_ is the fixed effect of genetic background *j*, *B*_*i*_ is a random effect for barcode *i*, and *ε*_*i*,*j*,*k*_ is the residual. We identified genes with significant fitness differences between genetic backgrounds by comparing the difference in the -2*log likelihoods of the models to a chi-square distribution with one degree of freedom, estimating a p-value, and accounting for multiple testing by controlling the false discovery rate [61]. We selected those genes with adjusted p-values less than 0.01. We took the contrast *T*_Δ*cdaA*_*−T*_*wt*_ to be the estimated fitness effect of knocking out the gene between the genetic backgrounds.

#### Sensitized interaction screen analysis

The sensitized-interaction screen involved six different starting pools (T_0_) of barcoded strains (3 with the control vector genetic background, 3 with a Δ*cdaA* genetic background). Each T_0_ pool was divided into constant light and LDC samples. We proceeded as above through log-2 transforming the sample-normalized number of reads. To account for different starting percentages of each barcode within the T_0_ pools, we subtracted the starting barcode values from the values after constant light or LDC growth for each sample. We also removed the 232 genes represented by fewer than 15 T_0_ reads in each pool, leaving 1,729 genes and 98,216 barcodes.

To determine a sensitized-interaction score for each gene, we used maximum likelihood to fit a pair of nested linear mixed effects models to the sample- and read-normalized log-2 transformed counts (S4 Dataset):

$$y_{i,j,k,m}=\mu_{g}+C_{j}+T_{m}+(C_{j}*T_{m})+B_{i}+\varepsilon_{i,j,k,m};B_{i}∼iid\;N\left(0,\varsigma_{g}{}^{2}\right);\varepsilon_{i,j,k,m}∼iid\;N\left(0,\sigma_{g}{}^{2}\right)$$ (3)

$$y_{i,j,k,m}=\mu_{g}+C_{j}+T_{m}+B_{i}+\varepsilon_{i,j,k,m};B_{i}∼iid\;N\left(0,\varsigma_{g}{}^{2}\right);\varepsilon_{i,j,k,m}∼iid\;N\left(0,\sigma_{g}{}^{2}\right)$$ (4)

where *y*_*i*,*j*,*k*,*m*_ is the normalized log-2 value for barcode *i* in gene *g* in condition *j* for sample *k* in genetic background *m*. *μ*_*g*_ is the average value for the gene; *C*_*j*_ is the fixed effect of condition *j* (either constant light or LDCs); *T*_*m*_ is a fixed effect of the genetic background (either wt or Δ*cdaA*); *B*_*i*_ is a random effect for barcode *i*; and *ε*_*i*,*j*,*k*,*m*_ is the residual. (Cj * Tm) represents the interaction between light condition and genetic background and estimates the effect in which we are interested—whether fitness difference for a strain between constant light and LDC conditions depends on the genetic background. By determining how well model (3), which includes the interaction term, fits the data compared to model (4), which does not, we can test whether the genetic background significantly affects the fitness difference. We identified genes for which model (3) fit significantly better than model (4) by comparing the difference in the -2*log likelihoods of the models to a chi-square distribution with one degree of freedom, estimating a p-value, and accounting for multiple testing as above by adjusting our p-values and accepting a false discovery rate of 0.01. We took the contrast (*C*_*LD*_*T*_Δ*cdaA*_*−C*_*LD*_*T*_*wt*_*)–(C*_*LL*_*T*_Δ*cdaA*_*—C*_*LL*_*T*_*wt*_*)* to estimate the fitness effects of the interaction between light condition and genetic background.

### Competition assays for validation of IRB-Seq

For one-on-one competition experiments single mutants were prepared for *cdaA* (AM5403) with Sp and Sm resistance, the putatively interacting mutants of interest carrying Km resistance, and WT controls for both Km resistance (8S15-K12), and Sp and Sm resistance (AM1303). Double mutants with the *cdaA* loss-of-function background and the putatively interacting second mutation were also prepared. The mutants were grown to OD_750_ ~ 0.4 and washed three times with BG-11 without antibiotics via centrifugation at 4,696 G for ten minutes and diluted to an OD of 0.015. Cultures were then mixed in a 1:1 ratio according to the desired competition being assayed and grown for 8 days under ~100 μmol photons·m^−2^·s^−1^.

Composition of competing populations was determined by leveraging the different antibiotic resistances of the strains. To assay survival of each genotype at zero, three, and eight days, mixed culture was serially diluted using a 1:10 dilution series over 6 wells. The dilutions were plated in 20 μL spots in duplicate onto ~2 mL of BG-11 agar with either Sp and Sm, or Km on a 24-well clear plate. Colony number from the dilution series across the two antibiotic regimes was used to calculate CFUs in the original competition culture. Differentiation of the CFUs for the double mutants (Km-, Sp and Sm-resistant) and single *cdaA* mutants (Sp- and Sm-resistant) was accomplished via subtraction of the CFUs growing on Km from those growing on Sp and Sm.

## Supporting information

S1 Fig Genotyping of the *cdaA* insertion mutant. On the left, the location of the transposon insertion mutation conferring Km resistance is shown over a schematic drawn to scale of the gene. On the right, the genotyping gel containing lanes: 1, amplification of *cdaA* mutant allele (8S16-L9), in which a 1.3 kb insertion is present, with primers surrounding the *cdaA* gene; 2, amplification of WT DNA with the same primers; 3, standard 1-kb ladder (New England BioLabs).
(PDF)

S2 Fig Mass spectrometry chromatograms of WT and *cdaA* mutant extracts. Each sample was mixed with an internal standard (heavy labeled c-di-AMP), detected as m/z 689 → 146 transition. Biological c-di-AMP was detected through four m/z transitions: 659 → 136 (as a qualifier and quantifier), 659 → 312 (as a qualifier), 659 → 330 (as a qualifier). (*A*) In WT extracts, all transitions corresponded well with the internal standard; (*B*) whereas in *cdaA* mutant extracts, only noise was detected.
(PDF)

S3 Fig Expression of *S*. *elongatus cdaA* in *E*. *coli*. The *S*. *elongatus cdaA* gene was expressed in a modified version of the IPTG inducible vector pMAL-c2X in DH5α (AM5466). Fold change is shown relative to uninduced vector. Error bars represent SE of two replicates.
(PDF)

S4 Fig Complementation of the *cdaA* mutant. The top panel shows the phenotype, measured by spot plate, of the *cdaA* mutant (8S16-L9) under constant light and LDCs. The bottom panel shows the phenotype of the *cdaA* mutant when a WT allele of the *cdaA* gene is added in trans to neutral site two (using vector AM5253). ***P<10^−3^.
(PDF)

S5 Fig Absorbance spectrum of *cdaA* mutant during a LDC. Mean absorbance values of the *cdaA* transposon mutant (8S16-L9) and WT at (*A*) 0 h, (*B*) 30 h, and (*C*) 36 h into an LDC. Absorbance is normalized to OD_750_ and each value represents the average of four replicates.
(PDF)

S6 Fig Genetic interaction with *flv1*. (*A*) Relative survival of the *cdaA* single mutant and the *cdaA-flv1* double mutant when grown competitively against each other. (*B*) Relative survival of WT and the *flv1* single mutant when grown competitively against each other. In all figure parts survival is determined by spot plates (see Materials and Methods) and error bars represent SE of three replicates.
(PDF)

S7 Fig Binding of c-di-AMP by KdpD. Binding of KdpD (Synpcc7942_1729) expressed in *E*. *coli* to c-di-AMP, determined by DRaCALA on cell lysate (see Materials and Methods). Error bars indicate SE of two replicates.
(PDF)

S8 Fig Assaying *rpoD2* for sensitized genetic interaction. (*A*) Relative survival of the *cdaA* single mutant and the *cdaA-rpoD2* double mutant when grown competitively against each other in constant light and LDCs. (*B*) Relative survival of WT and the *rpoD2* single mutant when grown competitively against each other in constant light and LDCs. Survival in all figure parts is determined by spot plates (see Materials and Methods) and error bars represent SE of three replicates.
(PDF)

S1 Dataset *cdaA* mutant genetic interactions. (XLSX)

S2 Dataset *cdaA* mutant sensitized genetic interactions. (XLSX)

S3 Dataset Interaction screen R script. This file contains the annotated R script for determining genetic interactions, where two genetic backgrounds within the library are compared. Also included are the files on which the script was run to produce interaction scores for the *cdaA* mutant: 1) “all.poolcount.txt”, the location and reads of barcoded transposon mutants for each sample; 2) “interaction_screen_exp.csv”, with designation of the samples as T_0_, control genotype, and experimental genotype; 3) “genes.tab”, the gene coordinate file used to map barcoded transposon mutants to genes.
(ZIP)

S4 Dataset Sensitized interaction screen R script. This file contains the annotated R script for determining sensitized genetic interactions, where two genetic backgrounds within the library are compared under two environmental conditions. Also included are the files on which the script was run to produce sensitized interaction scores for the *cdaA* mutant: 1) “Interaction_LD_all_pool.csv”, the location and reads of barcoded transposon mutants for each sample under LDCs; 2) “Interaction_LL_all_pool.csv”, the location and reads of barcoded transposon mutants for each sample under constant light; 3) “interaction_screen_exp.csv”, with designation of sample light regime, group (whether the samples are from the same or different T_0_s), and whether they are T_0_, control genotype, or experimental genotype; 4) “genes.tab”, the gene coordinate file used to map barcoded transposon mutants to genes.
(ZIP)
