# Supplementary figures and images for: High-throughput interaction screens illuminate the role of c-di-AMP in cyanobacterial nighttime survival
Source: PLoS Genet. 2018 Apr 2;14(4):e1007301. doi: 10.1371/journal.pgen.1007301 (PMC5897029; doi:10.1371/journal.pgen.1007301)

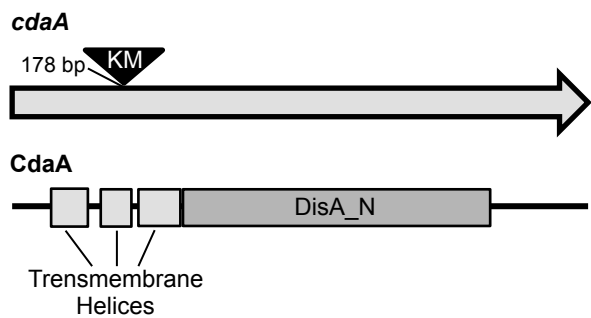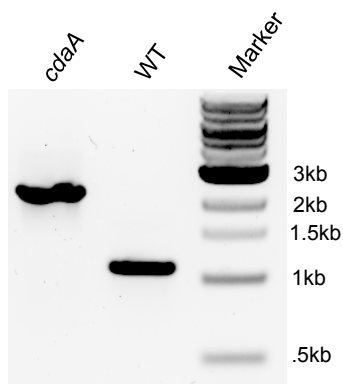

Supplement: S1 Fig — On the left, the location of the transposon insertion mutation conferring Km resistance is shown over a schematic drawn to scale of the gene. On the right, the genotyping gel containing lanes: 1, amplification of cdaA mutant allele (8S16-L9), in which a 1.3 kb insertion is present, with primers surrounding the cdaA gene; 2, amplification of WT DNA with the same primers; 3, standard 1-kb ladder (New England BioLabs). (PDF) [file pgen.1007301.s001.pdf]

**A**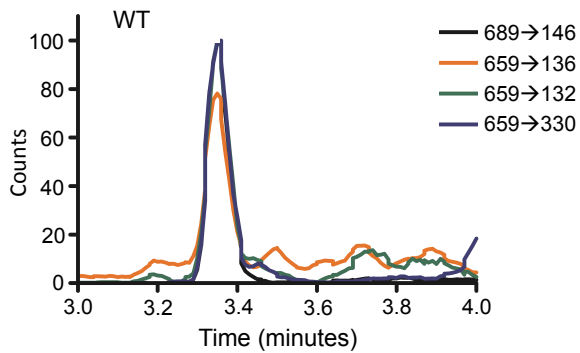**B**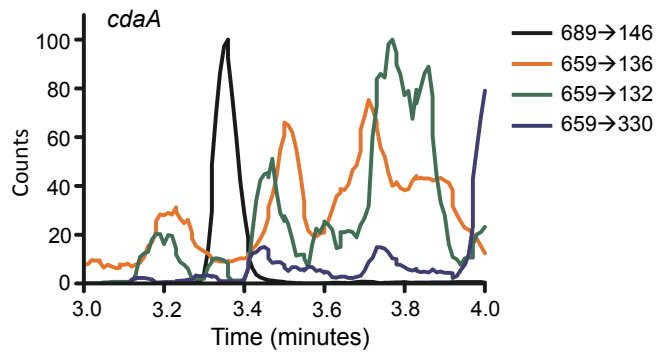

Supplement: S2 Fig — Each sample was mixed with an internal standard (heavy labeled c-di-AMP), detected as m/z 689 → 146 transition. Biological c-di-AMP was detected through four m/z transitions: 659 → 136 (as a qualifier and quantifier), 659 → 312 (as a qualifier), 659 → 330 (as a qualifier). (A) In WT extracts, all transitions corresponded well with the internal standard; (B) whereas in cdaA mutant extracts, only noise was detected. (PDF) [file pgen.1007301.s002.pdf]

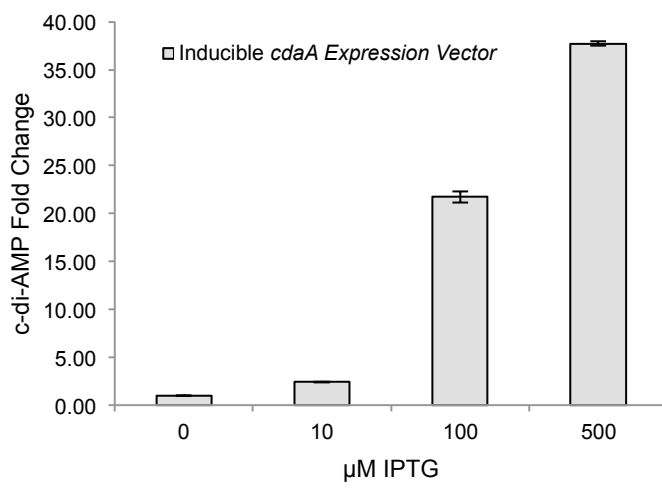

Supplement: S3 Fig — The S. elongatus cdaA gene was expressed in a modified version of the IPTG inducible vector pMAL-c2X in DH5α (AM5466). Fold change is shown relative to uninduced vector. Error bars represent SE of two replicates. (PDF) [file pgen.1007301.s003.pdf]

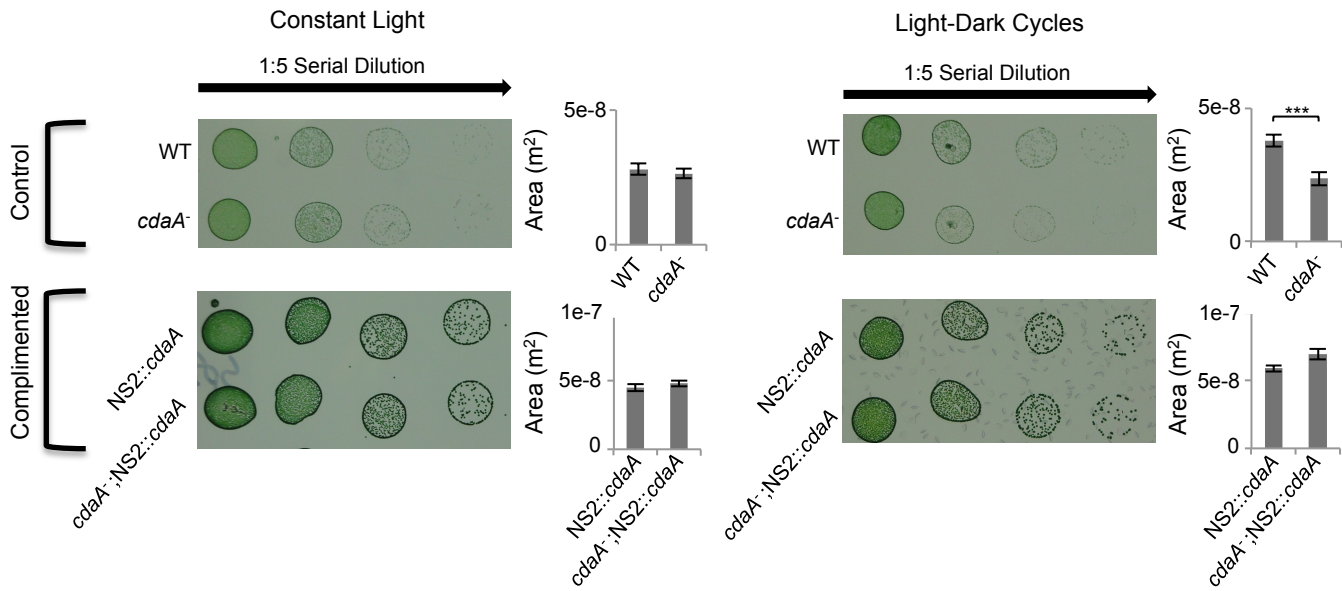

Supplement: S4 Fig — The top panel shows the phenotype, measured by spot plate, of the cdaA mutant (8S16-L9) under constant light and LDCs. The bottom panel shows the phenotype of the cdaA mutant when a WT allele of the cdaA gene is added in trans to neutral site two (using vector AM5253). ***P<10−3. (PDF) [file pgen.1007301.s004.pdf]

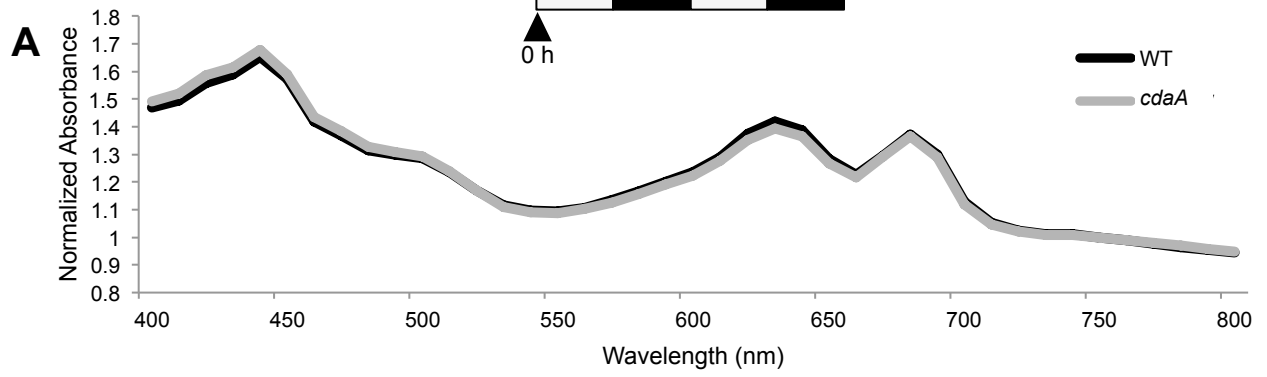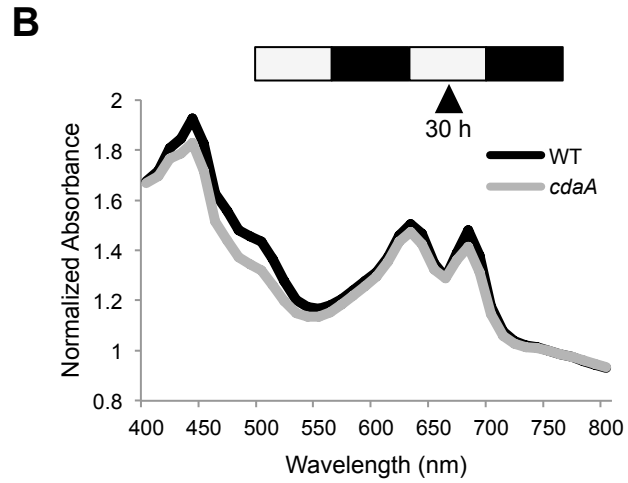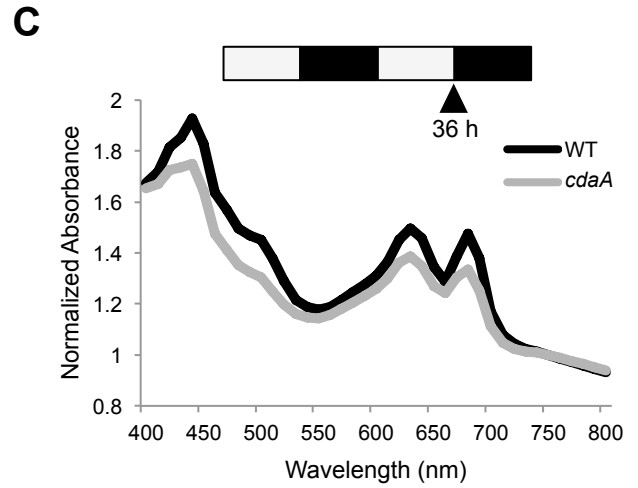

Supplement: S5 Fig — Mean absorbance values of the cdaA transposon mutant (8S16-L9) and WT at (A) 0 h, (B) 30 h, and (C) 36 h into an LDC. Absorbance is normalized to OD750 and each value represents the average of four replicates. (PDF) [file pgen.1007301.s005.pdf]

**A** Constant Light

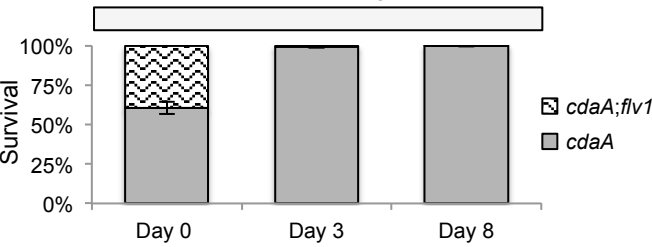

**B** Constant Light

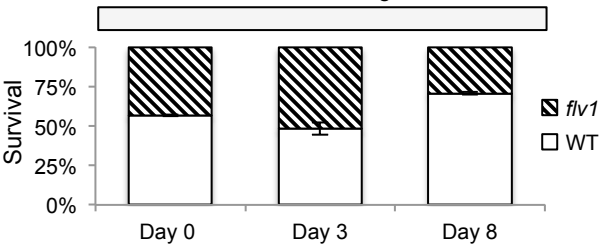

Supplement: S6 Fig — (A) Relative survival of the cdaA single mutant and the cdaA-flv1 double mutant when grown competitively against each other. (B) Relative survival of WT and the flv1 single mutant when grown competitively against each other. In all figure parts survival is determined by spot plates (see Materials and Methods) and error bars represent SE of three replicates. (PDF) [file pgen.1007301.s006.pdf]

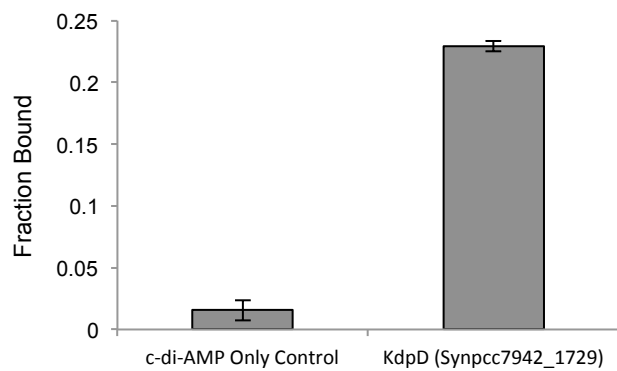

Supplement: S7 Fig — Binding of KdpD (Synpcc7942_1729) expressed in E. coli to c-di-AMP, determined by DRaCALA on cell lysate (see Materials and Methods). Error bars indicate SE of two replicates. (PDF) [file pgen.1007301.s007.pdf]

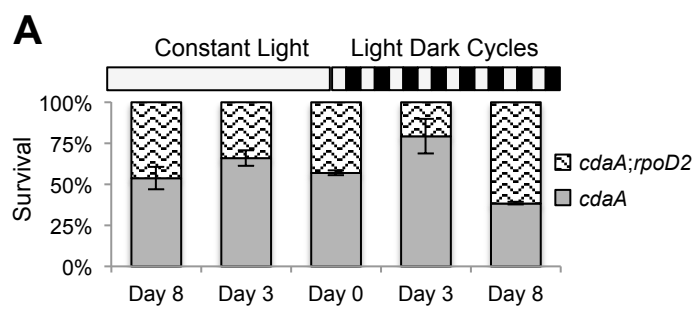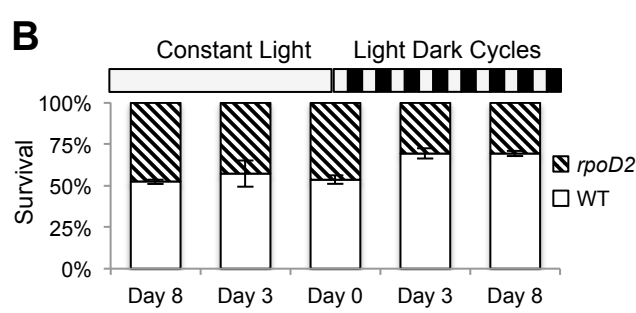

Supplement: S8 Fig — (A) Relative survival of the cdaA single mutant and the cdaA-rpoD2 double mutant when grown competitively against each other in constant light and LDCs. (B) Relative survival of WT and the rpoD2 single mutant when grown competitively against each other in constant light and LDCs. Survival in all figure parts is determined by spot plates (see Materials and Methods) and error bars represent SE of three replicates. (PDF) [file pgen.1007301.s008.pdf]
